# Artemether Ameliorates Non-Alcoholic Steatohepatitis by Repressing Lipogenesis, Inflammation, and Fibrosis in Mice

**DOI:** 10.3389/fphar.2022.851342

**Published:** 2022-05-02

**Authors:** Jia Xu, Xiaoyun He, Xianghui Huang, Feng Zhang, Xinxin Ren, Charles Asakiya, Yue Li, Kunlun Huang

**Affiliations:** ^1^ Key Laboratory of Precision Nutrition and Food Quality, Key Laboratory of Functional Dairy, Ministry of Education, College of Food Science and Nutritional Engineering, China Agricultural University, Beijing, China; ^2^ Key Laboratory of Safety Assessment of Genetically Modified Organism (Food Safety), The Ministry of Agriculture and Rural Affairs, Beijing, China; ^3^ Department of Pathology, Beijing Ditan Hospital, Capital Medical University, Beijing, China

**Keywords:** artemether, non-alcoholic steatohepatitis, inflammation, liver fibrosis, lipid metabolism, lipogenesis

## Abstract

**Background:** Non-alcoholic fatty liver disease (NAFLD) is a widespread disease, but no recognized drug treatment exists. Previous studies have shown that artemether (Art) can ameliorate carbon tetrachloride (CCl_4_)–induced liver fibrosis in mice. This study sets out to observe the therapeutic impact of Art on non-alcoholic steatohepatitis (NASH).

**Methods:** Model mice were provided with a methionine- and choline-deficient (MCD) diet for 4 weeks or a high-fat diet (HFD) for 28 weeks, respectively, and then treated with Art. RNA sequencing (RNA-Seq) analyzed gene expression changes caused by Art treatment. The molecular mechanism of the therapeutic effects of Art on NASH was studied in the mouse liver and HepG2 cells.

**Results:** Art treatment significantly attenuated hepatic lipid accumulation and liver damage in MCD diet– or HFD-induced NASH mice. The RNA-Seq analysis revealed lipid metabolism as a major pathway suppressed by Art administration, in addition to the regulation of inflammation pathways. Mechanistically, Art reduced lipid accumulation by repressing *de novo* lipogenesis of sterol regulatory element-binding protein-1c (SREBP-1c), acetyl-CoA carboxylase (ACC), fatty acid synthase (FASN), stearoyl-CoA desaturase (SCD1), promoting lipolysis of peroxisome proliferator–activated receptor-γ co-activator-1α (PGC1α), adipose triglyceride lipase (ATGL), and carnitine palmitoyltransferase I (CPT-1a) in NASH mouse liver and HepG2 cells. In addition, Art inhibited the secretion of pro-inflammatory factors and reduced inflammatory infiltration by effectively inhibiting M1 macrophage activation. Furthermore, Art inhibited transforming growth factor-beta 1 (TGF-β), and the SMAD signaling pathway mediates the development of liver fibrosis.

**Inclusion**: Art improved fat deposition by repressing *de novo* lipogenesis and promoting lipolysis *in vivo* and *in vitro*. Furthermore, Art improved inflammation and fibrosis with a significant effect. It is a prospective therapeutic agent for NASH.

## Introduction

Non-alcoholic fatty liver disease (NAFLD) is a pathologic syndrome that comprises non-alcoholic fatty liver (NAFL), non-alcoholic steatohepatitis (NASH), NASH-associated cirrhosis, and hepatocellular carcinoma (HCC). NAFLD is thought to be a hepatic demonstration of metabolic disorder and is often related to metabolic risk factors, such as obesity, dyslipidemia, hypertension, and diabetes (Chalasani et al., 2020). NAFLD is becoming a major chronic liver disease worldwide, with a worldwide prevalence of around 25% of the adult population, and an important cause of liver transplantation for primary hepatocellular carcinoma (Paik et al., 2020; Loomba et al., 2021).

The theory of multiple hits has already been proposed in NAFLD (Loomba et al., 2021). Fatty acids are a substrate for lipotoxic substances when oversupply or elimination is impaired, causing endoplasmic reticulum stress, hepatocellular injury, and death (Pei et al., 2020). It has been shown that the main causes of hepatic fatty acid oversupply include insulin resistance (IR) (Chen et al., 2019), increased hepatic *de novo* lipogenesis (DNL) (Lambert et al., 2014), and intrahepatic lipolysis defects (e.g., decreased ATGL/CGI-58 activity and decreased hepatic mitochondrial/peroxisome beta-oxidation) (Schweiger et al., 2009). Therefore, it is important to identify the source and clearance mechanism of fatty acids in hepatocytes to understand the metabolic basis of NASH and to identify therapeutic targets. Several drugs have previously been developed to inhibit fatty acid metabolism genes, such as pioglitazone, thiazolidinediones, and Aramchol. Unfortunately, there is no currently approved drug treatment for NAFLD (Violi and Cangemi, 2010; Iruarrizaga-Lejarreta et al., 2017).

Plant extracts or natural products have been extensively studied in preventing or improving NAFLD (Li et al., 2021). Artemether is a derivative of artemisinin and has good lipid solubility (Jung et al., 2004). Artemether is currently primarily used to treat malaria and attempts to treat a variety of malignancies. Guo Y et al. (2018) found that artemether could improve the degree of hepatic steatosis, glucose homeostasis, and insulin resistance in db/db mice, but they did not observe improvement in hepatic inflammation. Wang et al. (2019) showed that artemether had a certain repressive effect on liver fibrosis induced by CCl_4_ in mice, but the pathogenic mechanism of CCl_4_ was different from that of diet-induced NASH-associated fibrosis. However, there has been little discussion about the ameliorated effect of artemether on NASH, and its mechanism of action is unclear.

In this study, a NASH model induced by methionine- and choline-deficient (MCD) diet or high-fat diet (HFD) in mice and a HepG2 cell model were constructed, and the molecule mechanism of artemether in the protection of NASH was clarified.

## Materials and Methods

### Materials

Artemether was purchased from Solarbio, Beijing (SA8510, purity: HPLC ≥ 98%). A high-fat diet (60% of energy derived from fat) was purchased from Beijing Huafukang Co., Ltd. (H10060, Beijing, China). Methionine- and choline-deficient L-amino acid diet (MCD, 22% of calories derived from fat) and methionine- and choline-supplemented diet (MCS) were purchased from Trophic Animal Feed High-tech Co., Ltd., China (TP 3005GS and TP 3005G, Nantong, China).

### Animal Experiments

The animal program was approved by the Animal Ethics Committee of China Agricultural University (approval number: KY 1700025). Animal experiments were performed in the SPF Animal Room, Beijing Agricultural Product Quality Supervision, Inspection, and Testing Center of the Ministry of Agriculture. Six-week-old male C57BL/6J mice were purchased from Beijing Vital River Laboratory Animal Technology Co., Ltd., and fed in an acclimatization period of 1 week before the experiment.

**FIGURE 1:**
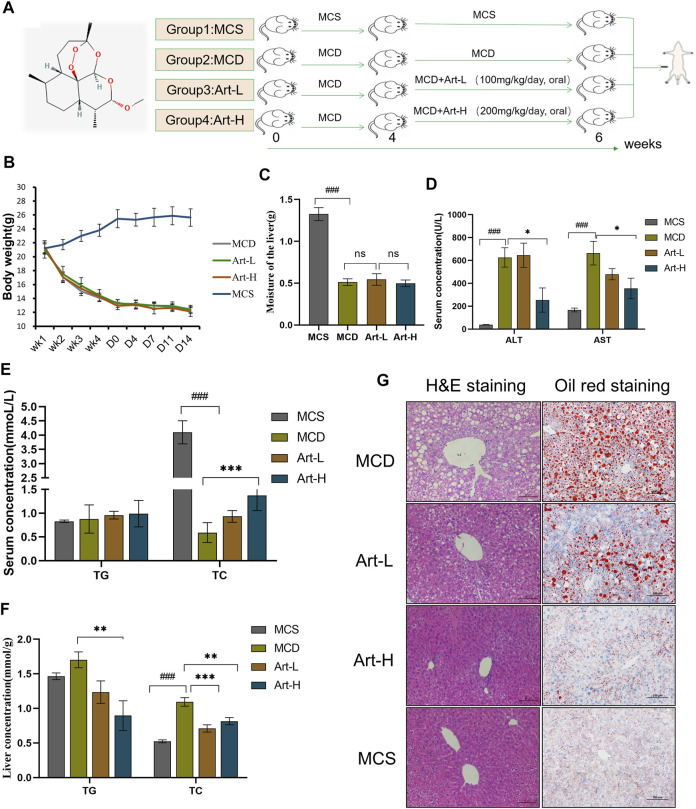
Effect of Artemether treatment on hepatic injury and hepatic steatosis in the MCD model. **(A)** Experimental design: after 4 weeks of the MCD diet-induced NASH model, the experiment was divided into four groups: the MCD diet group (MCD); MCD diet coupled with low-dose artemether (Art-L) group: 100 mg/kg BW; MCD diet coupled with high-dose artemether (Art-H) group: 200 mg/kg BW, artemether gavage once daily for 2 weeks; and MCS control group (MCS) (*n* = 6). **(B)** Body weight change after dosing in mice. **(C)** Mouse liver weight. **(D)** Serum ALT and AST levels in mice. **(E)** Serum TG and TC concentrations in mice. **(F)** TG and TC concentrations in the liver. **(G)** From left to right, H&E staining of the liver (scale bar: 50 μM) and oil red O staining of the liver (scale bar: 50 μM). The MCS was compared with the MCD group: ^#^ *p* < 0.05, ^##^ *p* < 0.01, and ^###^ *p* < 0.001. MCD compared with Art-L and Art-H groups: *p < 0.05, **p < 0.01, and ***p < 0.001.

The MCD diet-induced NASH model includes the following: 1) MCS group: mice were fed with MCS diet for 6 weeks; 2) MCD group: mice were fed with MCD diet, and 0.5% carboxymethylcellulose sodium (CMC-Na^+^) was given to MCS and MCD mice by oral administration; 3) Art-L and 4) Art-H group mice were fed MCD diet and orally administered with Art at 100 mg/kg or 200 mg/kg per day. MCD, Art-L, and Art-H groups maintained an MCD diet for 4 weeks and then were treated with artemether or vehicle for 2 weeks (Figure 1A).

**FIGURE 2:**
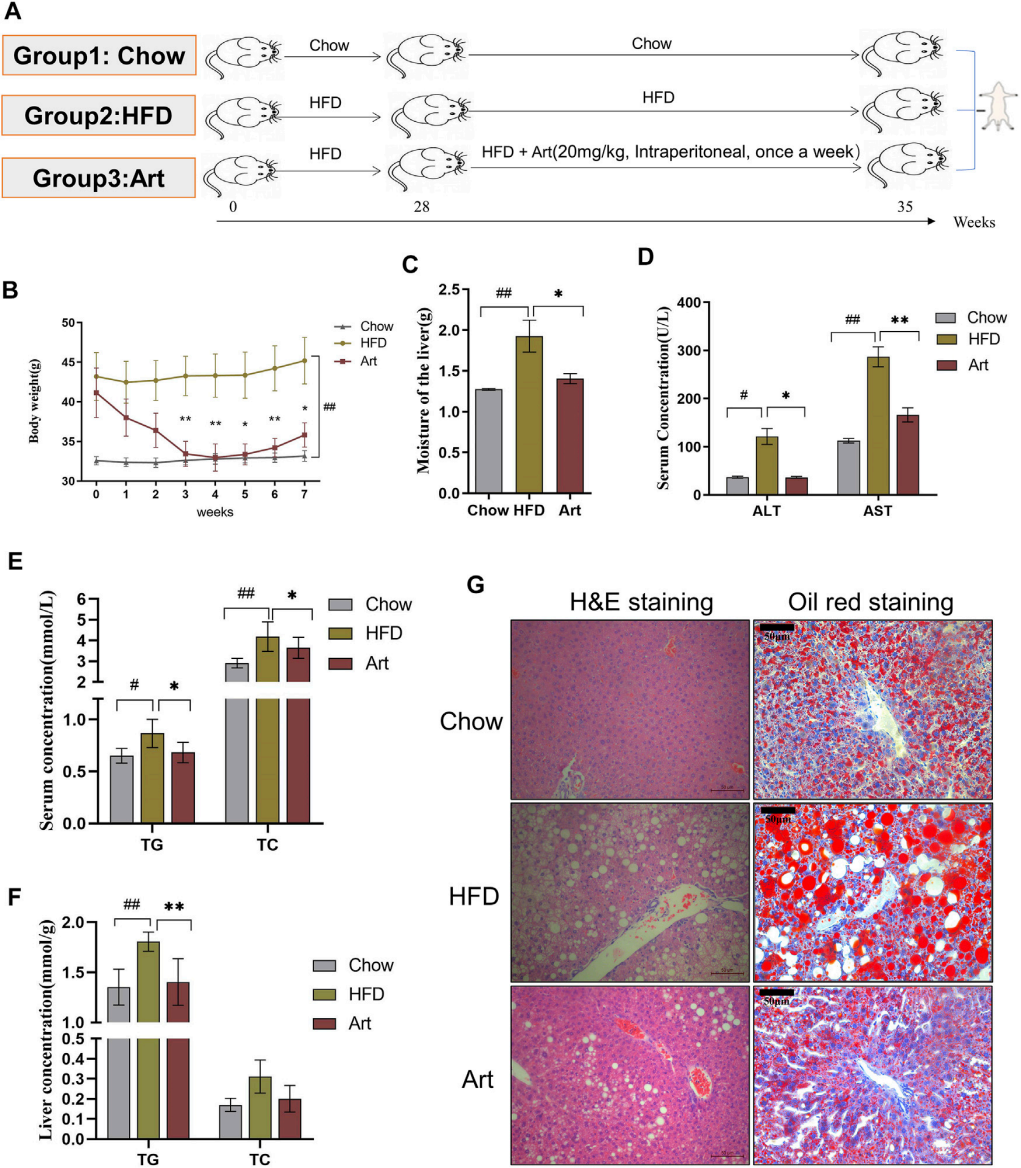
Artemether improves hepatic steatosis and liver injury in NASH mice induced by a HFD. **(A)** Experiment design was divided into three groups: chow diet group (Chow), high-fat diet group (HFD), and high-fat diet combined with the artemether treatment group (Art): 20 mg/kg BW intraperitoneally once a week for 7 weeks (*n* = 6). **(B)** Body weight change after dosing. **(C)** Liver weight. **(D)** Serum ALT and AST levels. **(E)** Serum TG and TC levels. **(F)** TG and TC concentrations in the liver. **(G)** From left to right, H&E staining of the liver (scale bar, 50 μM) and oil red O staining of the liver (scale bar: 50 μM). The Chow group vs. the HFD group: ^#^ *p* < 0.05, ^##^ *p* < 0.01, and ^###^ *p* < 0.001. The Art group compared with the HFD group: *p < 0.05, **p < 0.01, and ***p < 0.001.

The NASH model induced by HFD includes the following: 1) Normal diet group (chow): mice were fed with normal diet; 2) HFD mice group (HFD): mice were fed with HFD for 35 weeks, and DMSO was given to chow and HFD mice by intraperitoneal injection; 3) Artemether groups: mice were given HFD, and 20 mg/kg Art (dissolved in DMSO) was given by intraperitoneal injection. The HFD and artemether groups maintained an HFD for 28 weeks and were treated once a week for 7 weeks (Figure 2A).

Some of the livers of mice were fixed with 4% paraformaldehyde, and the rest were used for molecular and biochemical tests.

### Body Composition Measurements

The whole fat and lean masses of mice were detected with the NiuMag Small Animal Body Composition Analysis and Imaging System (MesoQMR 23-060H-I, Niumag Corp., Shanghai, China), according to the reference method.

### Determination of Glucose Tolerance in Mice

Fasting blood glucose was measured after 12 h fast. The glucose solution was administered intraperitoneally at 1.5 g/kg body weight, and blood glucose levels were detected at 15, 30, 60, 90, and 120 min after injection (Li et al., 2019).

### Determination of Biochemical Indications in Mice

Sera of mice were collected, and a biochemistry analyzer (98640000, Indiko™ Plus Clinical Chemistry Analyzer, Thermo Fisher Scientific, California, United States) was used to determine the levels of triglycerides (TG), cholesterol (TC), alkaline phosphatase (ALP), aspartate aminotransferase (AST), and alanine aminotransferase (ALT).

### RNA-Seq and Bioinformatic Analysis

TRIzol reagent (Thermo Fisher Scientific, Waltham, United States) was used to extract total RNA of HFD-fed mice liver. The mRNA library was constructed by Beijing Geek Gene Technology Co., Ltd. (Beijing, China) according to the NEBNext super RNA library preparation kit for Illumina. The main functions of differential genes were classified using the PANTHER database (HTTP://www.Un.Org). The enrichment of genes was carried out using the Metascape database (http://metascape.org/gp/index.html#/main/step1).

### Cell Viability Assay

HepG2 cells (purchased from Cell Resource Center, Institute of Basic Medicine, Chinese Academy of Medical Sciences) were seeded into 96-well plates and treated with 0.5 mM free fatty acids (oleic acid:palmitic acid 2:1, molar ratios) for 24 h with or without artemether, and cell viability was detected according to the protocol of the Cell Counting Kit-8 (C0038, Beyotime, Beijing, China).

### Liver Tissue and Cell Oil Red O Staining

In total, 4% paraformaldehyde-fixed liver samples were cryoprotected in 20% sucrose at 4°C overnight, and then, samples were embedded with OCT compounds in liquid nitrogen. Frozen blocks were sectioned with a cryostat (CM 1590, Leica, Wetzlar, Germany). The slides were fixed with 4% paraformaldehyde for 15 min, washed, dried, and stained with oil red dye solution (BA 4081, Baso Diagnostics, Zhuhai, China) (Rom et al., 2019). HepG2 cells were stimulated with 0.5 mM free fatty acids. Then, cells were treated with 6.25 and 25 μm artemether, respectively, for 24 h. Cells were fixed with 4% paraformaldehyde and then stained with oil red O solution (O0625, Sigma, Darmstadt, Germany), as described previously (Huang et al., 2019). The positive area of oil red O staining was quantified by ImageJ.

### Liver and Cellular Lipid Detection

The supernatant is taken after homogenization of the liver tissues; for HepG2 cells, the cell particles were collected in PBS buffer. The contents of TC and TG were determined with commercially available kits (A110-1-1 and A111-1-1, Nanjing Jiancheng Bioengineering Institute, Nanjing, China).

### Western Blot Analysis

For Western blot analysis, standard SDS-PAGE blotting methods were used. Primary antibodies used in Western blot are as follows: GAPDH, β-tubulin, TLR4, and MYD88 (Beyotime, Beijing, China); ATGL and PPAR-α (Santa Cruz Biotechnology, Inc., Dallas, United States); and CPT-1, SREBP-1c, FASN, ACC, SCD1, and NF-kB (Cell Signaling Technology, Danvers, MA, United States). Chemiluminescence was visualized using an imaging system (330037, Clinx Science Instruments Co. Ltd., Shanghai, China).

### Hematoxylin-Eosin, Masson, and Immunohistochemistry Staining

The fixed livers were dehydrated, embedded, sectioned, and then stained by hematoxylin-eosin (HE) and Masson’s staining (BA4079A, Zhuhai Beiso Biotech Co., Ltd.), and then, sections were observed under a microscope (DM2500, Leica, Germany) (Jia et al., 2019).

Slides were incubated with 3% hydrogen peroxide buffer and 10% normal goat serum. The primary antibodies included α-SMA, F4/80, CD11C, and CD206 (Cell Signaling Technology, Danvers, MA, United States) and TGF-β (Abcam, Waltham, United States), and secondary antibodies labeled with horseradish peroxidase were used. Detection was conducted using a horseradish peroxidase–based commercial detection system, disclosure with diaminobenzidine chromogen, and nuclear counterstaining with hematoxylin.

### Real-Time Quantitative PCR

RNA was reverse-transcribed into cDNA with a One-Step gDNA Removal and cDNA Synthesis SuperMix (AT311, TransGen Biotech, Beijing, China). Real-time quantitative PCR (qPCR) was performed with a SuperReal PreMix Plus (FP 205-03, TIANGEN Biotech, Beijing, China) by using a real-time PCR system (C1000, Bio-Rad, California, United States). Gene expression levels were normalized to β-actin. Primer sequences are listed in Supplementary Table S1.

### Statistical Analysis of Data

Statistical analyses were performed using GraphPad Prism 8 (GraphPad Software Inc., La Jolla, CA, United States). The significance of the difference between groups was calculated by Student’s unpaired t-test or one-way ANOVA (Tukey’s multiple comparison tests). Significant differences were considered when *p* < 0.05. Data are presented as means ± standard deviation (SD).

## Results

### Artemether Improved Liver Injury and Lipid Deposition in the Methionine- and Choline-Deficient Diet–Induced Non-Alcoholic Steatohepatitis Model

First, we assayed the effects of artemether on MCD-induced liver injury and steatosis in NASH mice. Mice were divided into four groups, two of which were fed with MCD for 4 weeks and treated with Art-L (100 mg/kg) and Art-H (200 mg/kg) for 2 weeks, respectively (Figure 1A). The results showed that the body weight decreased by about 50% after 6 weeks of MCD treatment compared with that of the MCS group, whereas artemether at 100 and 200 mg/kg had no significant effect on the body weight of MCD mice (Figure 1B). Similarly, wet liver weight decreased by 75% in the MCD group, but no significant difference between the MCD and Art group was observed (Figure 1C). The serum ALT and AST of the MCD group were significantly higher than those of the MCS group, and artemether could significantly reverse this phenomenon. The reversal effect of the Art-H (200 mg/kg) group was better than that of the Art-L (100 mg/kg) group (Figure 1D). The serum lipid assay showed that treatment with Art-H (200 mg/kg) significantly reversed the trend of decrease in serum TC due to the MCD diet but had no effect on TG levels (Figure 1E).

We also observed a reversal effect of artemether on hepatic steatosis induced by the MCD diet in NASH mice. We found that treatment with Art-H (200 mg/kg) significantly reduced TG and TC levels (Figure 1F). In addition, H&E staining showed that numerous hepatocytes in the MCD group appeared as vacuole-like steatosis with inflammatory cell infiltration. In contrast, the number and area of fatty vacuoles and inflammatory cell infiltration in liver tissues of artemether-treated mice were decreased, and the pathological morphology of liver of MCD mice was improved in a dose-dependent mode (Figure 1G). Oil red O staining also demonstrated a significant increase in lipid deposition in the MCD mouse liver, and the treatment with artemether reduced lipid accumulation in a dose-dependent mode (Figure 1G).

### Artemether Improved Hepatic Steatosis and Liver Injury in the HFD-Induced Non-Alcoholic Steatohepatitis Model

We also tested the impact of artemether on liver injury and steatosis in NASH mice induced by an HFD because MCD mice differed from human NASH in their pathogenesis. As shown in Figure 2A, we found that artemether could decrease the body weight of the HFD by 20% (Figure 2B). Compared with the Chow group, the percentage of fat in the HFD group was significantly increased by 2.05 times (*p* < 0.05), while that in the Artemether group was significantly decreased by 56.4% (Supplementary Figure.S1A). GTT analysis showed that treatment with artemether significantly improved glucose intolerance in mice. (Supplementary Figures.S1B,C). In the HFD group, the wet liver weight was significantly increased by 1.5 times compared with that of the control group (*p* < 0.01). However, artemether treatment reduced the wet liver weight by 27.1% (*p* < 0.05) (Figure 2C). Compared with the Chow group, the level of serum ALT and AST in the HFD group increased 3.3 times and 2.5 times, respectively. Compared with the HFD group, serum ALT decreased by 70% and AST decreased by 42% in the artemether group (*p* < 0.05) (Figure 2D). The analysis of TG and TC concentrations in serum showed that artemether significantly reversed the increase of the serum lipid level induced by the HFD (*p* < 0.05) (Figure 2E).

Similarly, we observed the anti-hepatic steatosis effect of Art in NASH mice induced by the HFD. In the HFD group, the levels of hepatic TG were increased by 1.34 times as compared with those of the control group (*p* < 0.01). However, artemether treatment reduced the hepatic TG concentration by 22.2% (*p* < 0.01). Artemether had no significant effect on hepatic TC in mice (Figure 2F). H&E staining showed that a large number of hepatocytes in the HFD group showed vacuolar steatosis with inflammatory infiltration, and artemether improved the pathological morphology of the liver in the HFD group. The number and area of fat vacuoles were significantly decreased, and the accumulation of inflammatory infiltration was decreased in the HFD group (Figure 2G). Oil red O staining confirmed that compared with the Chow group, lipid accumulation was significantly increased in the HFD group and decreased in the artemether group (Figure 2G). In conclusion, artemether could effectively reverse liver damage and hepatic steatosis induced by the HFD.

### Artemether Influences the Lipid Metabolism Pathway in the Liver of Non-Alcoholic Steatohepatitis Mice

**FIGURE 3:**
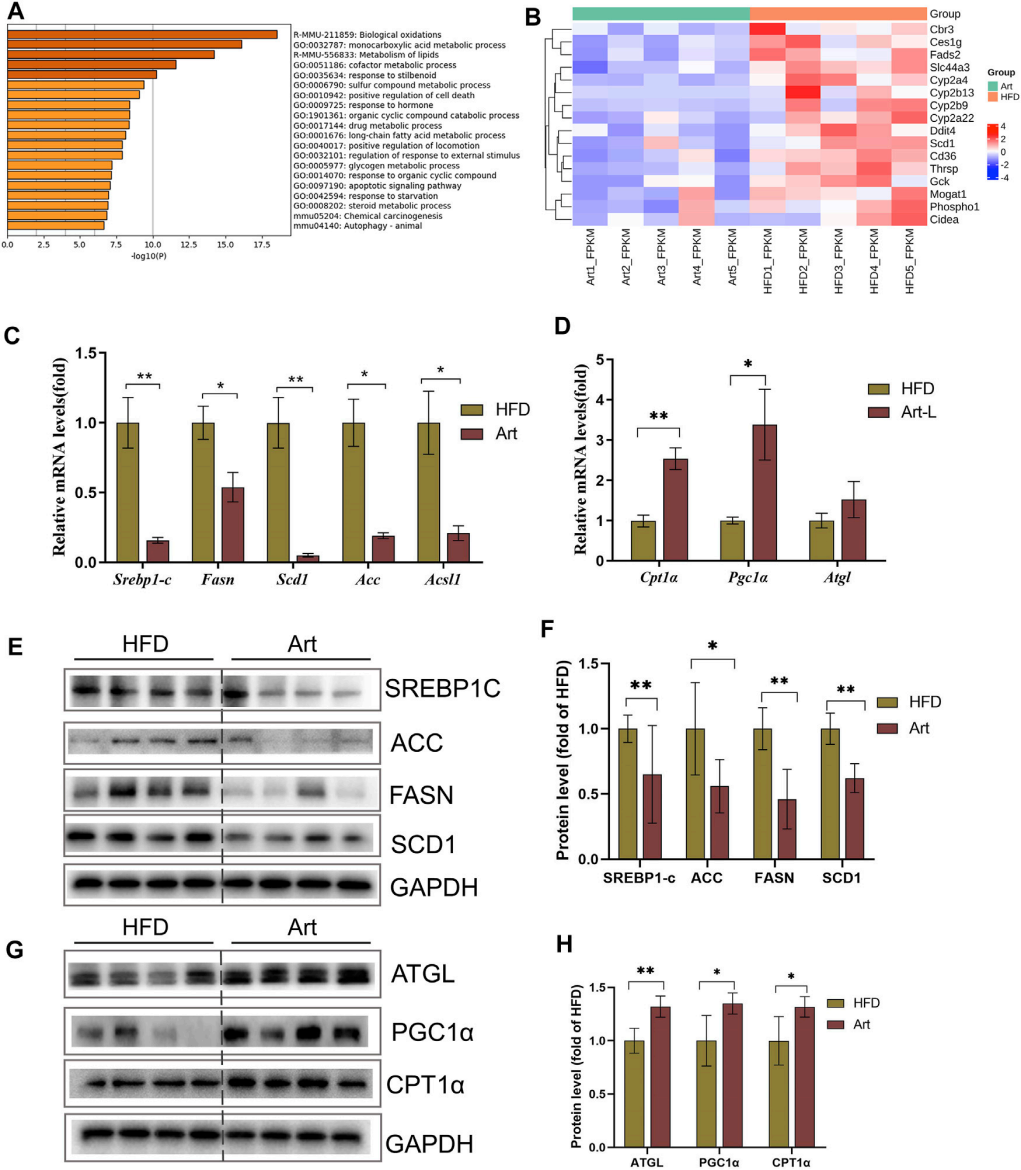
Effect of artemether treatment on the liver lipid metabolic pathway in NASH mice induced by an HFD. **(A)** KEGG pathway enrichment of the differential gene. **(B)** Heat map of the free fatty acid metabolism-related gene. **(C,D)** Real-time PCR analysis of lipid synthesis-related genes **(C)** and lipolysis-related genes **(D)** in the liver. **(E–H)** Expression and quantification analysis of lipid synthesis-related protein **(E,F)** and lipid breakdown-related protein **(G,H)** in the liver were detected by western blot. *p < 0.05, **p < 0.01, and ***p < 0.001.

To explore the molecular mechanism by which artemether ameliorated liver pathological phenotypes in NASH mice, RNA-Seq was used to analyze the liver transcriptome in NASH models induced by the HFD and in Art-treated mice. Volcanic mapping of differentially expressed genes (DEGs) revealed the whole change in gene expression patterns between HFD and Artemether-treated mice. A total of 1713 DEGs were selected from Artemether-treated mice, of which 883 were elevated and 830 were decreased (Supplementary Figures.S2A,B). DEGs are classified by the PANTHER database, and involved molecular functions including molecular regulation, transcriptional regulation, catalysis, and translation regulation. The biological processes include metabolism and stimulation (Supplementary Figure.S2C). DEGs were enriched using the Metascape database, with the most prominent metabolic pathways in the first 20 pathways being monocarboxylic acid metabolism, lipid metabolism, and long-chain fatty acid metabolism. In addition, enriched pathways include inflammation, insulin response, and protein folding (Figure 3A). We analyzed significantly downregulated genes of the artemether group, compared with the HFD group, which were mainly enriched in lipid metabolism pathways (Supplementary Figure.S2D). Artemether reduced fatty acid synthesis and transport-related gene expressions, including *Cbr3*, *Ces1g*, *Fads 2*, *Slc44a3*, *Cyp2a4*, *Cyp2b13*, *Cyp2b9*, *Cyp2a22*, *Ddit4*, *Scd1*, *Cd36*, *Thrsp*, *Gck*, *Mogat 1*, *Phospho1*, and *Cieda* (Figure.3B).

### Artemether Improved Hepatic Steatosis in Non-Alcoholic Steatohepatitis Mice by Repressing *De Novo* Lipogenesis and Promoting Lipolysis

Excessive TG storage in the liver is a dynamic imbalance between lipogenesis and lipolysis. SCD1, a key gene for *de novo* lipogenesis, was decreased in RNA-Seq assays. Therefore, we first analyzed whether artemether could improve hepatic lipid metabolism by inhibiting DNL. Interestingly, treatment with artemether significantly reduced the expression of the key genes involved in *de novo* lipogenesis, containing *Srebp-1c*, *Fasn*, *Acc*, *Scd1*, and *Ascl* (Figure 3C). Western blotting further confirmed that artemether decreased the protein expressions of SREBP-1C, ACC, FASN, and SCD1 in the liver tissue (Figures 3E,F).

Second, we considered the effects of artemether on lipolysis and fatty acid β-oxidation-related gene expressions. The results showed that artemether significantly increased the expression of fatty acid β-oxidation genes, including *Pgc1α* and *Cpt1α* (Figure 3D). The protein levels of ATGL, PGC-1a, and CPT-1α were also confirmed by Western blotting (Figures 3G,H). However, artemether had no significant effect on lipid synthesis genes (*Fasn*, *Acc*, and *Scd1*) in the MCD model and significantly increased the expression of lipolysis-related genes *Hsl*, *Mcad*, *Atgl*, and *Cpt1α.* (Supplementary Figures S3A,B). Therefore, artemether primarily reduced hepatic lipid deposition by increasing the expression of lipolysis genes in NASH mice induced by the MCD diet.

### Artemether Reduced Oleic-Palmitic Acid–Stimulated Lipid Deposition and Cellular Damage in HepG2 Cells

**FIGURE 4:**
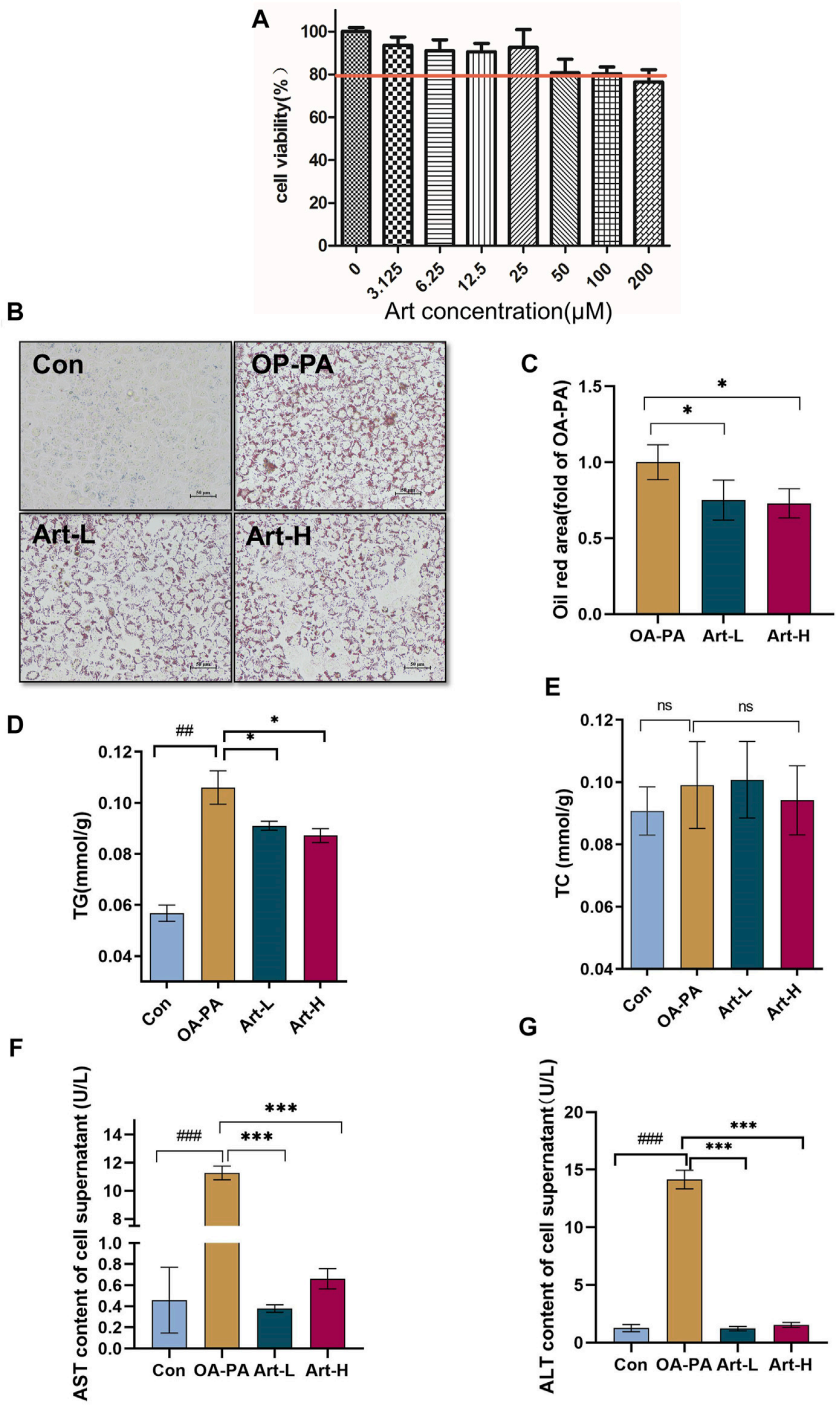
Artemether treatment reduces lipid deposition in oleic and palmitic acid–treated cells. **(A)** Cytotoxicity effect of artemether treatment in the of HepG2 cells (*n* = 6). **(B)** Images of oil red O staining of HepG2 cells that were treated with BSA (Con), 0.5 mM FFA mixture (OA-PA), 6.25 μM Art along with 0.5 mM FFA (Art-L), and 25 μM Art along with 0.5 mM FFA mixture (Art-H). **(C)** Quantification of oil red O staining with ImageJ. **(D)** Intracellular triglyceride level. **(E)** Intracellular cholesterol level. **(F,G)** AST **(F)** and ALT **(G)** levels in the supernatant of HepG2 cells. (*n* = 3). Con vs. OA-PA: ^#^ *p* < 0.05; ^##^ *p* < 0.01, and ^###^ *p* < 0.001. OA-PA compared with Art-L and Art-H: ^*^ *p* < 0.05, ^**^ *p* < 0.01, and ^***^ *p* < 0.001.

Next, we observed the improved effect of artemether on hepatic lipid deposition and explored its mechanism in oleic acid (OA):palmitic acid (PA)–induced fatty liver models of HepG2 cells. CCK-8 analysis showed that the concentrations of 6.25 μmol/L and 25 μmol/L artemether had no toxic effect on HepG2 cells, so we used these concentrations as the working concentrations in the following experiments (Figure 4A). Art-L (6.25 μmol/L) and Art-H (25 μmol/L) significantly decreased the area of oil red O staining, respectively (*p* < 0.05) (Figures 4B,C). The detection of the intracellular TG concentration showed that artemether significantly reduced the intracellular TG concentration in a dose-dependent mode (*p* < 0.05) (Figure 4D). There was no evidence that revealed artemether influences the intracellular TC content (Figure 4E). Artemether significantly reduced the AST and ALT levels in the supernatant of HepG2 cells (*p* < 0.001) (Figures 4F,G).

### Artemether Improved Lipid Metabolism of Oleic-Palmitic Acid–Stimulated HepG2 Cells by Repressing *De Novo* Lipogenesis and Promoting Lipolysis

**FIGURE 5:**
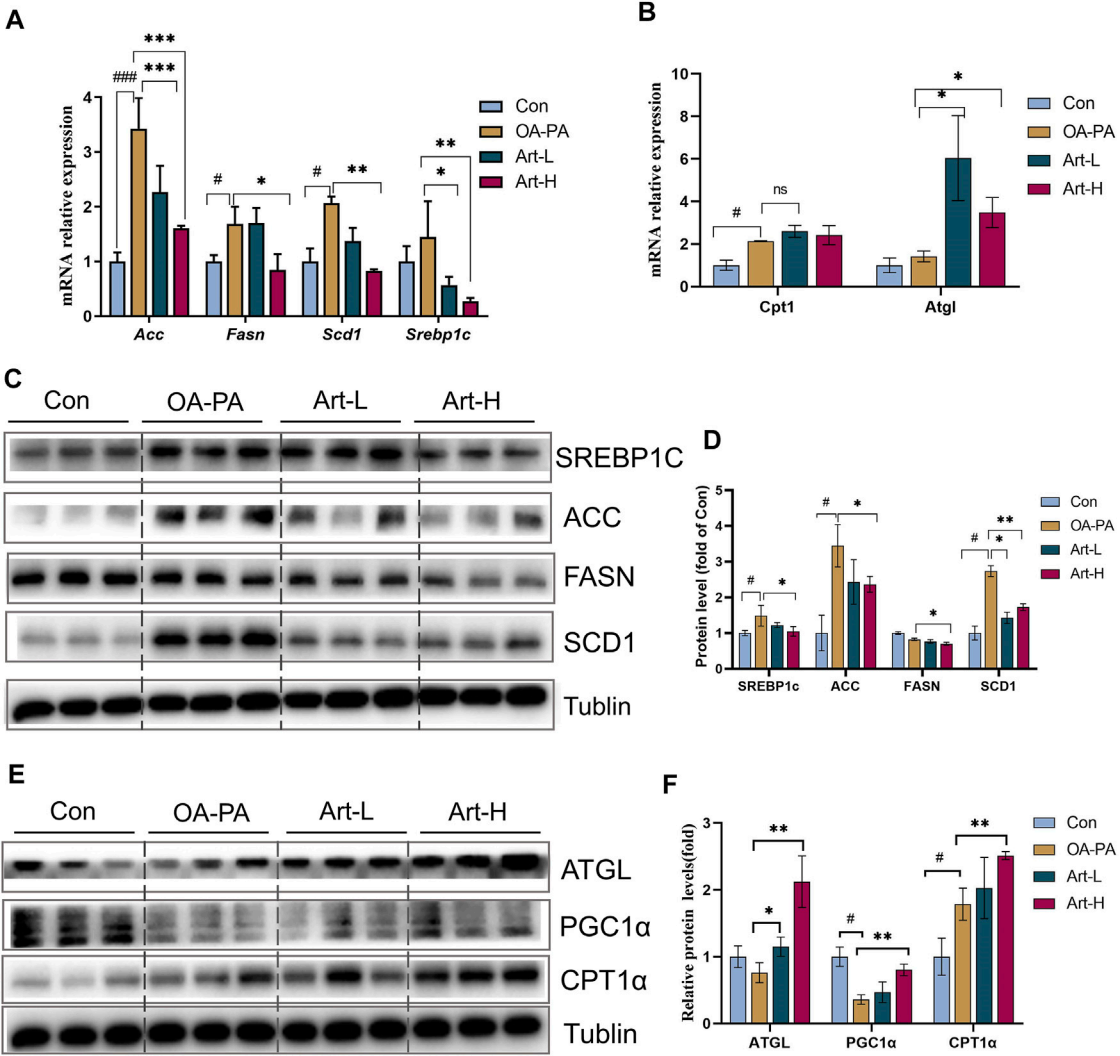
Artemether improves lipid metabolism induced by oleic and palmitic acids in HepG2 cells. **(A,B)** Real-time PCR analysis of lipid synthesis-related genes **(A)** and lipolysis-related genes **(B)** in the HepG2 cells. **(C)** Western blot was used to detect the expression of lipid synthesis-related proteins in HepG2 cells. **(D)** Quantitative plots of the protein expression associated with lipid synthesis. **(E)** Western blot detected the expression of lipolytic protein in HepG2 cells (*n* = 3). **(F)** Quantitative plots of lipid breakdown-related protein expression. Con vs. OA-PA: ^#^ *p* < 0.05, ^##^ *p* < 0.01, and ^###^ *p* < 0.001. OA-PA vs. Art-L and Art-H: *p < 0.05, **p < 0.01, and ***p < 0.001.

The mechanism of artemether improving lipid metabolism was further validated in HepG2 cells. The results of mRNA analysis showed that artemether inhibited the gene transcription of *Acc*, *Fasn*, *Scd1*, and *Srebp-1* significantly (Figure 5A). The proteins of SREBP-1C, ACC, FASN, and SCD1 were also confirmed by Western blotting (Figures 5C,D). In addition, artemether significantly increased the transcription expression of *Atgl* (Figure 5B). At the protein level, the expressions of ATGL and PGC-1a were significantly increased by artemether treatment (Figures 5E,F). These results demonstrated that artemether significantly improved hepatic fatty deposition by inhibiting the expression of key enzymes of DNL and increasing lipolysis in HepG2 cells.

### Artemether Reduced Hepatic Inflammation in High-Fat Diet-Induced Non-Alcoholic Steatohepatitis Mice

**FIGURE 6:**
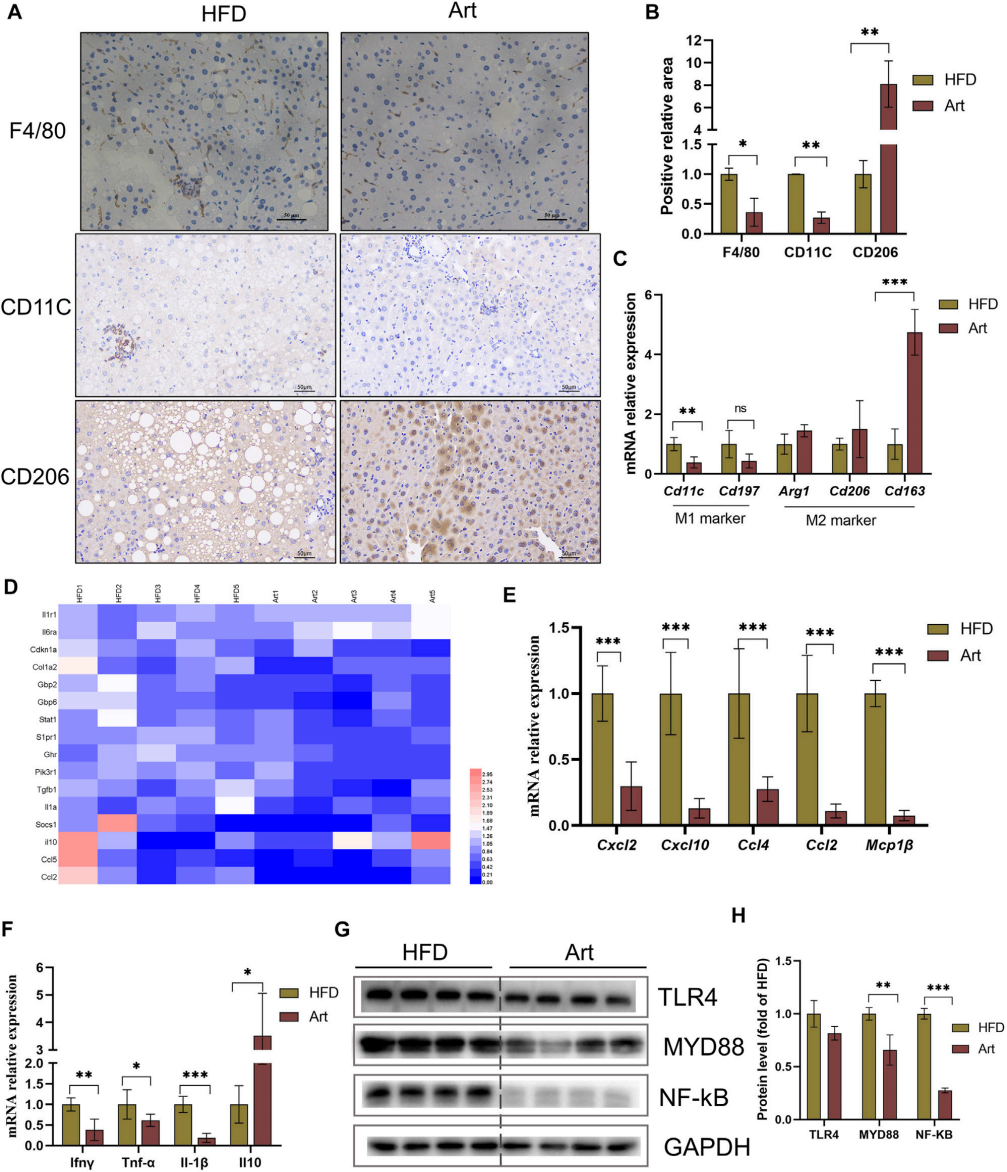
Artemether reduced HFD-induced liver inflammation. **(A)** F4/80, CD11C, and CD206 immunohistochemistry. **(B)** Quantification of F4/80, CD11C, and CD206 staining positive areas (*n* = 3). **(C)** qRT-PCR was used to detect the expression of M1-type marker genes (*Cd11* and *Cd197*) and M2-type marker genes (*Arg1*, *Cd206*, and *Cd163*). **(D)** Heat map of liver inflammation–related differential genes between NASH and Art groups. **(E,F)** Real-time PCR analysis of chemokine-related genes **(E)** and inflammatory factor-related genes **(F)** in the liver. **(G,H)** Protein levels of TLR4, MYD88, and NF-kb in the liver. *p < 0.05, **p < 0.01, and ***p < 0.001.

Liver inflammation is an important pathological feature of NASH, so we also observed the effect of artemether on liver inflammation in mice with NASH. Immunohistochemical staining of F4/80, CD11C, and CD206 showed that artemether significantly decreased liver inflammation by inhibiting M1-type macrophage activation and increasing M2-type polarization (Figures 6A,B). qPCR analysis showed that artemether significantly decreased *Cd11c* mRNA levels and significantly increased *Cd163* mRNA levels (Figure 6C). Enrichment analysis of hepatic transcriptional pathways also revealed that some of the genes downregulated by artemether treatment were closely related to the inflammatory pathway (Figure 2B), including interleukin-4 and interleukin-13 signaling (*Stat 1*, *Pik3r1*, *Socs1*, *Ccl2*, *Col1a2*, *Cdkn1a*, *S1pr1*, *Fos*, *Il6ra*, *Il10*, *Il1a*, and *Tgfb1*). (Figure 6D). qPCR analysis further confirmed that artemether significantly inhibited the expressions of chemokines (*Ccl4*, *Ccl2*, *Cxcl12*, *Cxcl10*, and *Mcp1β*) and pro-inflammatory genes (*Ifnγ*, *IL-1β*, and *TNF-α*) (Figures 6E,F) and significantly increased the expression of the anti-inflammatory gene (*Il10*) (Figure 6F). Western blotting showed that artemether decreased the levels of TLR4 and significantly decreased the levels of Myd88 and NF-κB (Figures 6G,H).

### Artemether Ameliorated the High-Fat Diet-Induced Liver Fibrosis

**FIGURE 7:**
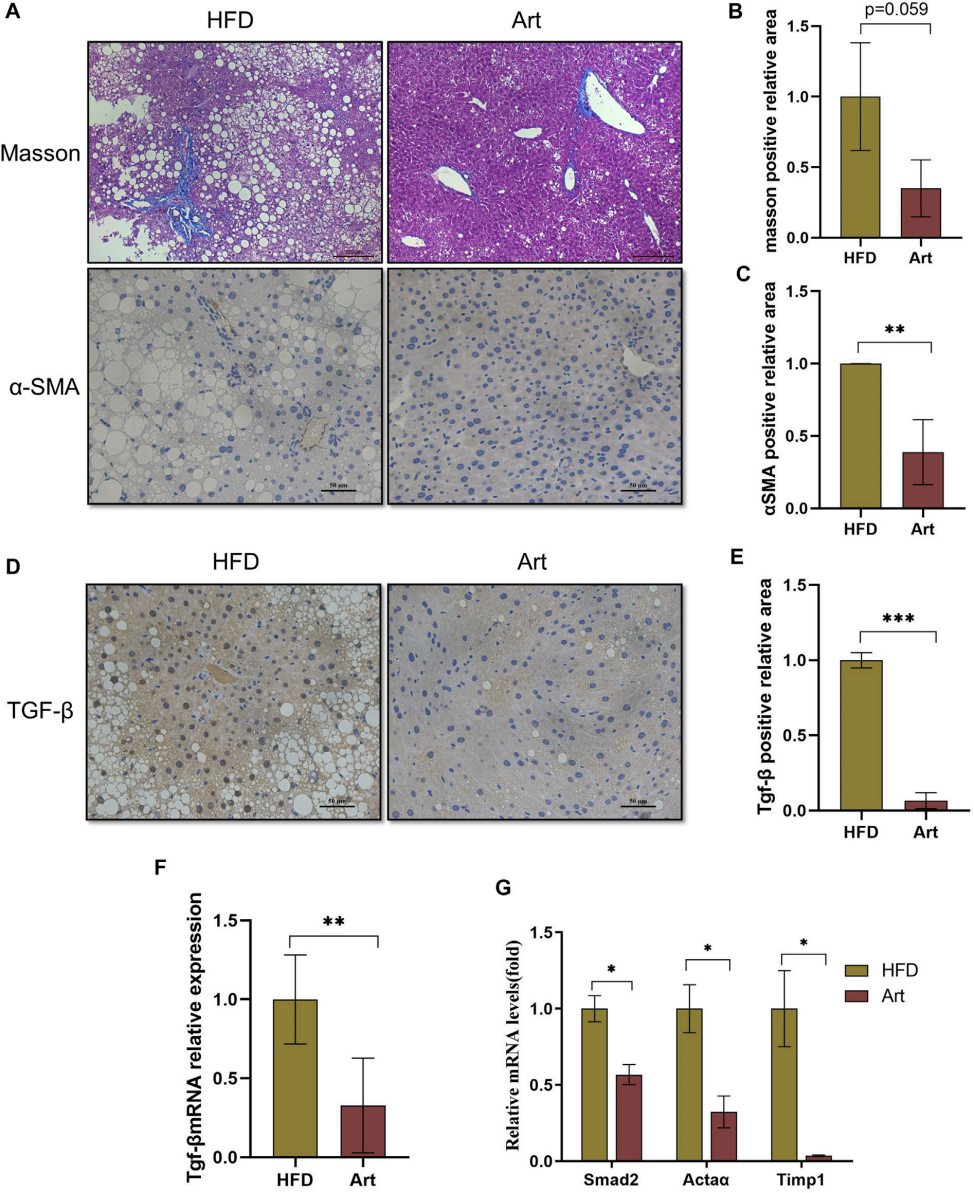
Artemether prevented NASH-induced liver fibrosis. **(A)** From top to bottom: Masson’s staining (scale bar: 50 μM) and α-SMA immunohistochemistry (scale bar: 50 μM). **(B)** Quantification of Masson’s staining area. **(C)** Quantification of immunohistochemical staining for α-SMA. **(D)** TGF-β immunohistochemical staining (scale bar: 50 μM). **(E)** Quantification of TGF-β immunohistochemical staining. **(F,G)** Real-time PCR analysis of hepatic TGF-β expression **(F)** *Acta 2*, *Timp1*, and *Smad2* **(G)** in the liver. *p < 0.05, **p < 0.01, and ***p < 0.001.

Previous research has shown that artemether prevents CCl_4_-induced liver fibrosis in mice (Wang et al., 2019). Does artemether improve liver fibrosis in NASH model mice? Masson staining and immunohistochemical analysis of α-smooth muscle actin (α-SMA) showed that artemether had a significant inhibitory effect on NASH-related liver fibrosis (Figures 7A–C). It is well known that the TGF-β/SMAD pathway mediates the development of liver fibrosis, of which TGF-β1 is the key initiator of fibrosis (Xu et al., 2016). Therefore, we examined whether artemether could reduce TGFβ-mediated liver fibrosis. These results showed artemether significantly decreased the *TGF-β* gene expression (Figure 7F) and protein level (Figures 7D,G). Finally, we tested other genes in the SMAD pathway and showed that the treatment with artemether significantly decreased fibrogenic gene expressions in the NASH mouse liver, including *smad2*, *Timp1*, and *Actaα* (Figure 7H).

## Discussion

Although NAFLD disease is becoming more prevalent worldwide, the main challenge faced by many researchers is the lack of authorized drugs specifically for the treatment of NAFLD (Paik et al., 2020). Screening and exploring novel therapeutic agents have an important social and scientific value (Peng et al., 2020). Artemether was found for the first time to improve hepatic steatosis, inflammatory infiltrates, and fibrotic progression in mice induced by HFD and MCD diet. Molecular mechanistic studies based on RNA-Seq analysis and cell models suggested that artemether plays a major role in repressing *de novo* lipogenesis, promoting lipolysis, and increasing fatty acid beta-oxidation.

Monomeric analysis of Chinese herbal drugs is considered one of the effective approaches for exploiting new drugs. In addition to the treatment of malaria, recently, investigators have examined the effects of artemether on the treatment of metabolic disorders. Artemether has improved glucose metabolism in a diabetic mouse model (Chalasani et al., 2020). Kim et al. (2016) found that *Artemisia annua* leaf extract administration in Sprague–Dawley rats with HFD-induced obesity, the major source of artemisinin, prevented the development of liver fibrosis and reduced lipid storage and inflammation in the liver. Guo Y et al. (2018) found that artemether improves glucose metabolism abnormalities in db/db mice by reducing pancreatic β-cell apoptosis and increasing insulin emission in mice. However, it did not explore the impact on liver inflammation and fibrosis in mice. This study constructed two well-known mouse models of NASH using the HFD and MCD diet. The results demonstrated that artemether consistently significantly improved the degree of hepatic steatosis, inflammatory injury, and fibrotic progression in the mouse model. In the MCD model, positive concentration-related treatment effects were also observed.

In this study, artemether could significantly inhibit the weight gain and reduce the body fat rate of NASH mice induced by the HFD, which showed a good effect on weight loss. In addition, artemether significantly improved glucose tolerance in NASH mice. The large accumulation of subcutaneous and visceral fat in obese individuals can induce insulin resistance and liver inflammation (Loomba et al., 2021), so improving obesity and weight loss is one of the important strategies for the treatment of NASH. These results reflect those of Lu et al. (2016), who also found that artemether injected subcutaneously or intravenously *via* the tail vein could effectively reduce the weight gain induced by a high-fat diet improve cold tolerance and insulin sensitivity in mice. Furthermore, the study conducted by Guo Y et al. (2018) showed that artemether significantly reduced body weight and improved insulin sensitivity in db/db mice. Subsequently, we will investigate whether artemether reduces hepatic lipid deposition by improving adipose tissue inflammation and insulin resistance.

One of the more significant findings to emerge from this study is that DNL synthesis key genes, including ACC, SREBP-1C, FASN, and SCD1, were significantly inhibited in Art-treated mice and hepatocyte models. These results further demonstrated that artemether, as a natural inhibitor of the DNL pathway, decreases hepatic lipid deposition primarily by inhibiting the DNL pathway. In this study, RNA-Seq screening in HFD-induced NASH confirmed that regulating lipid metabolism-related pathways is the primary pathway for artemether. DNL synthesis is mainly mediated by ACC, FASN, and SCD1, which play a critical role in promoting hepatic lipid deposition. Of these, ACC is an essential enzyme in the DNL process. In a phase II trial, the treatment of high-dose ACC inhibitors for 12 weeks significantly reduced hepatic steatosis in patients with NASH, but elevated serum TG levels were considered likely to be due to a compensatory increase in SREBP-1C activity (Kim et al., 2017). In our study, the expression of ACC in the liver was decreased, and the serum TG concentration was decreased in mice. SCD1 is a rate-limiting enzyme for hepatocyte lipogenesis. Aramchol, the inhibitor of SCD1, reduces hepatic steatosis in mice (Iruarrizaga-Lejarreta et al., 2017). SREBP-1c is an important transcription factor upstream of hepatic TG synthesis, which plays an important role in regulating the FASN expression and increasing lipid synthesis (Friedman et al., 2018). Our results demonstrate that treatment with artemether significantly inhibits the expression of SREBP-1C and its downstream FASN.

We also found that artemether could act as a potential drug to modulate macrophage polarization to reduce inflammatory damage in NASH. Hepatic macrophages have an obvious inflammatory phenotype that promotes disease progression in NASH through a variety of mechanisms (Tacke, 2017). The inflammatory microenvironment induces macrophages to polarize into the pro-inflammatory M1 type, increases the secretion of pro-inflammatory cytokines (TNF-α and IL-1β) and chemokines (CCL2 and CCL4), inhibits the polarization of M2-type macrophages, decreases the secretion of anti-inflammatory cytokines (IL-10), and increases the disproportionality of M1/M2-type macrophages (Li et al., 2020). In this study, artemether treatment significantly decreased the expression of the M1 macrophage marker genes (*Cd11c* and *Cd197*), pro-inflammatory factors (*TNF-α* and *IL-1β*), and chemokines (*Ccl4*, *Ccl2*, and *Mcp1*). At the same time, the expression of M2-type macrophage markers (*Arg-1* and *Cd206*) and anti-inflammatory factor (*Il-10*) were significantly increased by artemether treatment. There is scope for further progress in determining the relationship between artemether and metabolic inflammation. Guo C et al. (2018) found that the SCAP-SREBP2 complex incorporates NLRP3 inflammasome activation and cholesterol biosynthetic signaling during inflammation. We also found that artemether administration greatly decreased the cholesterol levels of liver. Further work could examine more closely the links between cholesterol and the SCAP-SREBP2 pathway by artemether administration in the NASH model.

**FIGURE 8:**
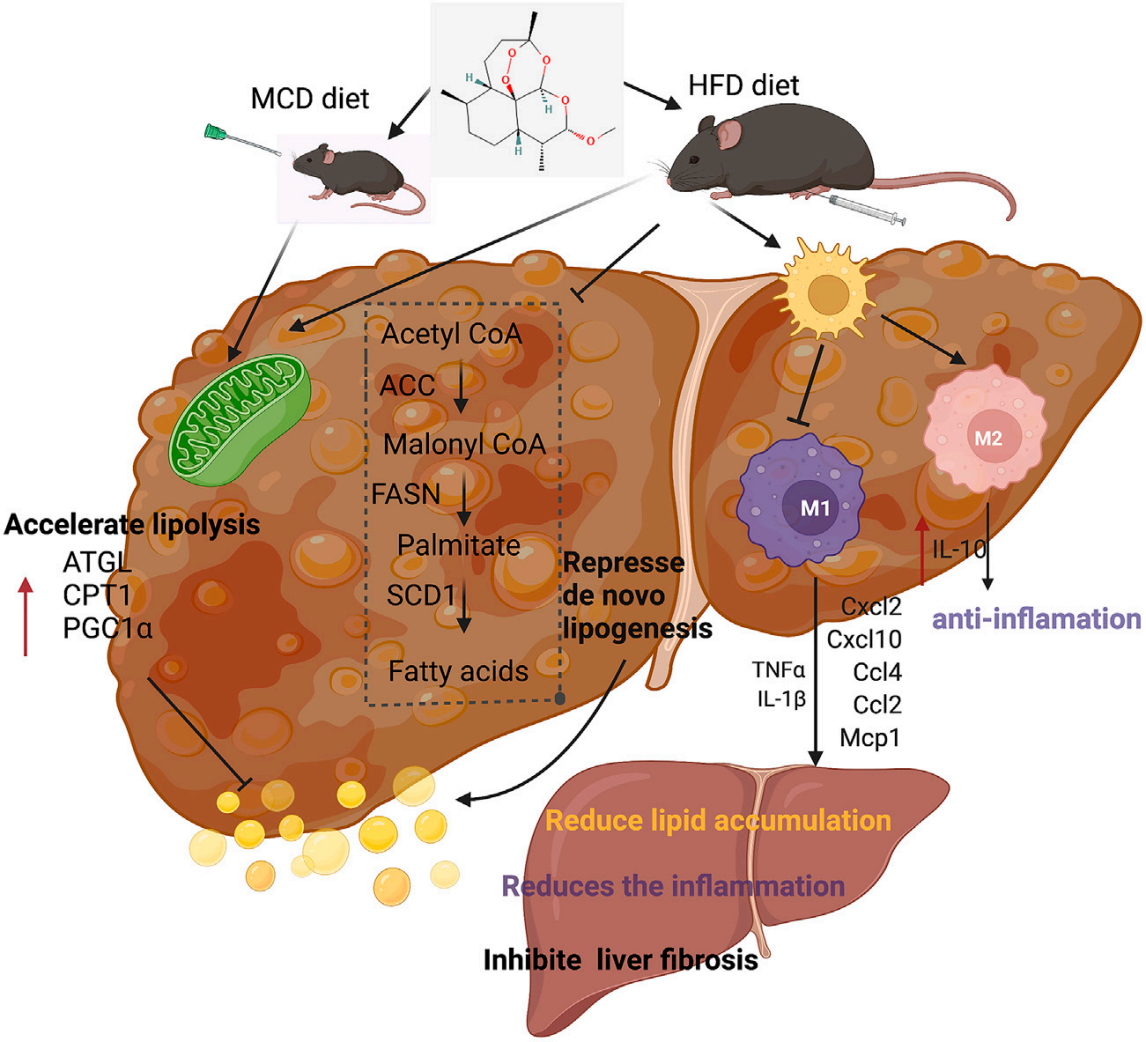
Summary figure showing that the presumable molecular mechanism of artemether ameliorates non-alcoholic steatohepatitis.

In conclusion, our experiments showed the therapeutic effects of artemether on NASH and related disorders. Artemether effectively reduced lipid deposition by repressing *de novo* lipogenesis and promoting lipid breakdown. In addition, artemether inhibited the secretion of pro-inflammatory factors and reduced inflammatory infiltration by promoting the polarization of M2 macrophages in livers. Furthermore, artemether inhibited the TGF-β/SMAD pathway and mediates the development of liver fibrosis. Finally, our discovery provided a theoretical reference for artemether use in clinical studies (Figure 8).

## Data Availability

The datasets presented in this study can be found in online repositories. The names of the repository/repositories and accession number(s) can be found below: Sequence Read Archive (SRA), PRJNA803877.

## References

[B1] Chalasani N. P. Ramasubramanian T. S. Bhattacharya A. Olson M. C. Edwards V. D. K. Roberts L. R. (2020). A Novel Blood-Based Panel of Methylated DNA and Protein Markers for Detection of Early-Stage Hepatocellular Carcinoma. Clin. Gastroenterol. Hepatol. 19, 2597–2605. 10.1016/j.cgh.2020.08.065 32889146

[B2] Chen Z. Yu Y. Cai J. Li H. (2019). Emerging Molecular Targets for Treatment of Nonalcoholic Fatty Liver Disease. Trends Endocrinol. Metab. 30, 903–914. 10.1016/j.tem.2019.08.006 31597607

[B3] Friedman S. L. Neuschwander-Tetri B. A. Rinella M. Sanyal A. J. (2018). Mechanisms of NAFLD Development and Therapeutic Strategies. Nat. Med. 24, 908–922. 10.1038/s41591-018-0104-9 29967350 PMC6553468

[B4] Guo C. Chi Z. Jiang D. Xu T. Yu W. Wang Z. (2018a). Cholesterol Homeostatic Regulator SCAP-SREBP2 Integrates NLRP3 Inflammasome Activation and Cholesterol Biosynthetic Signaling in Macrophages. Immunity 49, 842. 10.1016/j.immuni.2018.08.021 30366764

[B5] Guo Y. Fu W. Xin Y. Bai J. Peng H. Fu L. (2018b). Antidiabetic and Antiobesity Effects of Artemether in Db/db Mice. Biomed. Res. Int. 2018, 8639523. 10.1155/2018/8639523 29862294 PMC5971258

[B6] Huang Z. Wu L. M. Zhang J. L. Sabri A. Wang S. J. Qin G. J. (2019). Dual Specificity Phosphatase 12 Regulates Hepatic Lipid Metabolism through Inhibition of the Lipogenesis and Apoptosis Signal-Regulating Kinase 1 Pathways. Hepatology 70, 1099–1118. 10.1002/hep.30597 30820969 PMC6850665

[B7] Iruarrizaga-Lejarreta M. Varela-Rey M. Fernández-Ramos D. Martínez-Arranz I. Delgado T. C. Simon J. (2017). Role of Aramchol in Steatohepatitis and Fibrosis in Mice. Hepatol. Commun. 1, 911–927. 10.1002/hep4.1107 29159325 PMC5691602

[B8] Jia Q. Cao H. Shen D. Li S. Yan L. Chen C. (2019). Quercetin Protects against Atherosclerosis by Regulating the Expression of PCSK9, CD36, PPARγ, LXRα and ABCA1. Int. J. Mol. Med. 44, 893–902. 10.3892/ijmm.2019.4263 31524223 PMC6658003

[B9] Jung M. Lee K. Kim H. Park M. (2004). Recent Advances in Artemisinin and its Derivatives as Antimalarial and Antitumor Agents. Curr. Med. Chem. 11, 1265–1284. 10.2174/0929867043365233 15134519

[B10] Kim K. E. Ko K. H. Heo R. W. Yi C. O. Shin H. J. Kim J. Y. (2016). Artemisia Annua Leaf Extract Attenuates Hepatic Steatosis and Inflammation in High-Fat Diet-Fed Mice. J. Med. Food 19, 290–299. 10.1089/jmf.2015.3527 26741655 PMC4799707

[B11] Kim C. W. Addy C. Kusunoki J. Anderson N. N. Deja S. Fu X. (2017). Acetyl CoA Carboxylase Inhibition Reduces Hepatic Steatosis but Elevates Plasma Triglycerides in Mice and Humans: A Bedside to Bench Investigation. Cell Metab. 26, 394. 10.1016/j.cmet.2017.07.009 28768177 PMC5603267

[B12] Lambert J. E. Ramos-Roman M. A. Browning J. D. Parks E. J. (2014). Increased De Novo Lipogenesis Is a Distinct Characteristic of Individuals with Nonalcoholic Fatty Liver Disease. Gastroenterology 146, 726–735. 10.1053/j.gastro.2013.11.049 24316260 PMC6276362

[B13] Li H. Zhang C. Liu J. Xie W. Xu W. Liang F. (2019). Intraperitoneal Administration of Follistatin Promotes Adipocyte browning in High-Fat Diet-Induced Obese Mice. PLoS One 14, e0220310. 10.1371/journal.pone.0220310 31365569 PMC6668797

[B14] Li C. L. Zhou W. J. Ji G. Zhang L. (2020). Natural Products that Target Macrophages in Treating Non-alcoholic Steatohepatitis. World J. Gastroenterol. 26, 2155–2165. 10.3748/wjg.v26.i18.2155 32476782 PMC7235205

[B15] Li H. Y. Gan R. Y. Shang A. Mao Q. Q. Sun Q. C. Wu D. T. (2021). Plant-Based Foods and Their Bioactive Compounds on Fatty Liver Disease: Effects, Mechanisms, and Clinical Application. Oxid. Med. Cel Longev. 2021, 6621644. 10.1155/2021/6621644 PMC7939748 33728021

[B16] Loomba R. Friedman S. L. Shulman G. I. (2021). Mechanisms and Disease Consequences of Nonalcoholic Fatty Liver Disease. Cell 184, 2537–2564. 10.1016/j.cell.2021.04.015 33989548 PMC12168897

[B17] Lu P. Zhang F. C. Qian S. W. Li X. Cui Z. M. Dang Y. J. (2016). Artemisinin Derivatives Prevent Obesity by Inducing browning of WAT and Enhancing BAT Function. Cell Res. 26, 1169–1172. 10.1038/cr.2016.108 27633061 PMC5113303

[B18] Paik J. M. Golabi P. Younossi Y. Srishord M. Mishra A. Younossi Z. M. (2020). The Growing Burden of Disability Related to Nonalcoholic Fatty Liver Disease: Data from the Global Burden of Disease 2007‐2017. Hepatol. Commun. 4, 1769–1780. 10.1002/hep4.1599 33305148 PMC7706296

[B19] Pei K. Gui T. Kan D. Feng H. Jin Y. Yang Y. (2020). An Overview of Lipid Metabolism and Nonalcoholic Fatty Liver Disease. Biomed. Res. Int. 2020, 4020249. 10.1155/2020/4020249 32733940 PMC7383338

[B20] Peng C. Stewart A. G. Woodman O. L. Ritchie R. H. Qin C. X. (2020). Non-Alcoholic Steatohepatitis: A Review of its Mechanism, Models and Medical Treatments. Front. Pharmacol. 11, 603926. 10.3389/fphar.2020.603926 33343375 PMC7745178

[B21] Rom O. Xu G. Guo Y. Zhu Y. Wang H. Zhang J. (2019). Nitro-fatty Acids Protect against Steatosis and Fibrosis during Development of Nonalcoholic Fatty Liver Disease in Mice. EBioMedicine 41, 62–72. 10.1016/j.ebiom.2019.02.019 30772307 PMC6444056

[B22] Schweiger M. Lass A. Zimmermann R. Eichmann T. O. Zechner R. (2009). Neutral Lipid Storage Disease: Genetic Disorders Caused by Mutations in Adipose Triglyceride lipase/PNPLA2 or CGI-58/ABHD5. Am. J. Physiol. Endocrinol. Metab. 297, E289–E296. 10.1152/ajpendo.00099.2009 19401457

[B23] Tacke F. (2017). Targeting Hepatic Macrophages to Treat Liver Diseases. J. Hepatol. 66, 1300–1312. 10.1016/j.jhep.2017.02.026 28267621

[B24] Violi F. Cangemi R. (2010). Pioglitazone, Vitamin E, or Placebo for Nonalcoholic Steatohepatitis. N. Engl. J. Med. 363, 1185–1186. 10.1056/NEJMc1006581 20857543

[B25] Wang L. Zhang Z. Li M. Wang F. Jia Y. Zhang F. (2019). P53-dependent Induction of Ferroptosis Is Required for Artemether to Alleviate Carbon Tetrachloride-Induced Liver Fibrosis and Hepatic Stellate Cell Activation. IUBMB Life 71, 45–56. 10.1002/iub.1895 30321484

[B26] Xu F. Liu C. Zhou D. Zhang L. (2016). TGF-β/SMAD Pathway and its Regulation in Hepatic Fibrosis. J. Histochem. Cytochem. 64, 157–167. 10.1369/0022155415627681 26747705 PMC4810800

