# Supplementary figures and images for: Artemether Ameliorates Non-Alcoholic Steatohepatitis by Repressing Lipogenesis, Inflammation, and Fibrosis in Mice
Source: Front Pharmacol. 2022 May 2;13:851342. doi: 10.3389/fphar.2022.851342 (PMC9108288; doi:10.3389/fphar.2022.851342)

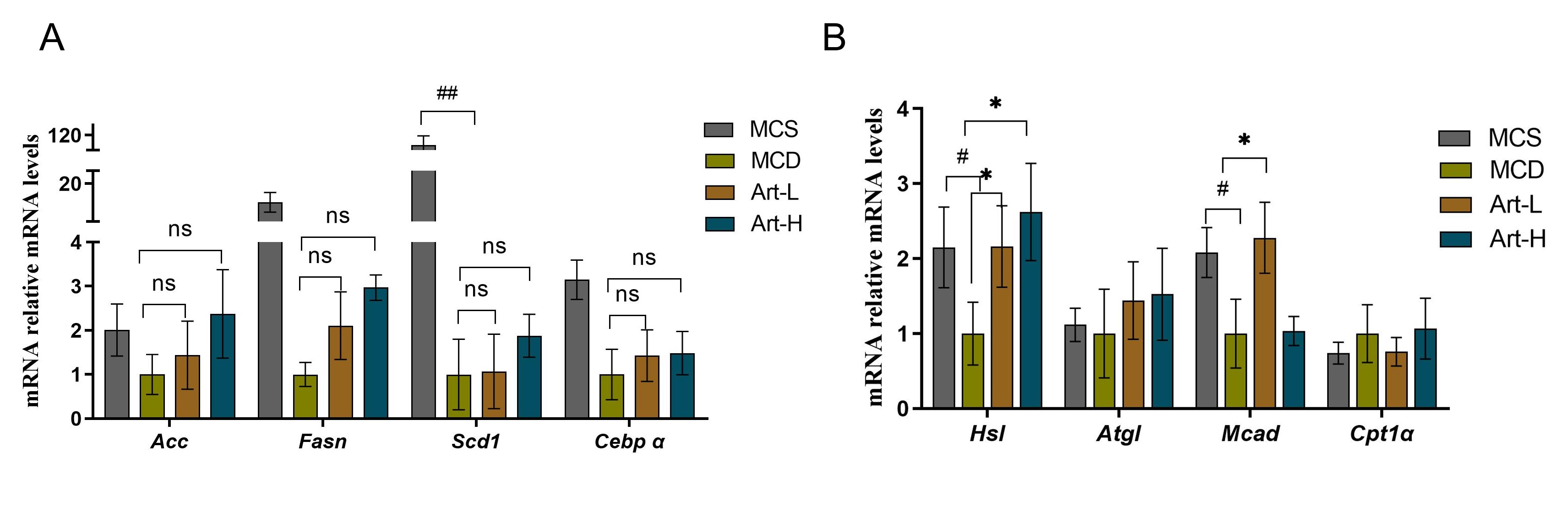

Supplement: Supplementary file 1 [file Image3.JPEG]

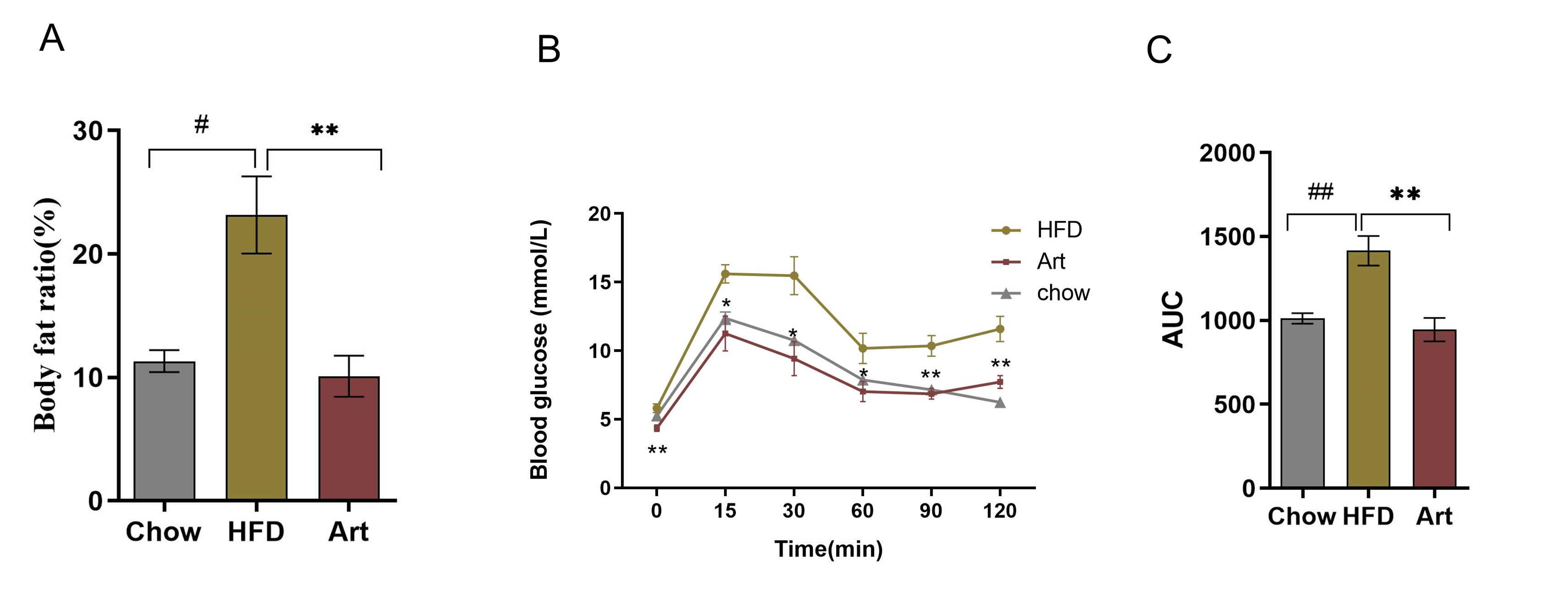

Supplement: Supplementary file 2 [file Image1.JPEG]

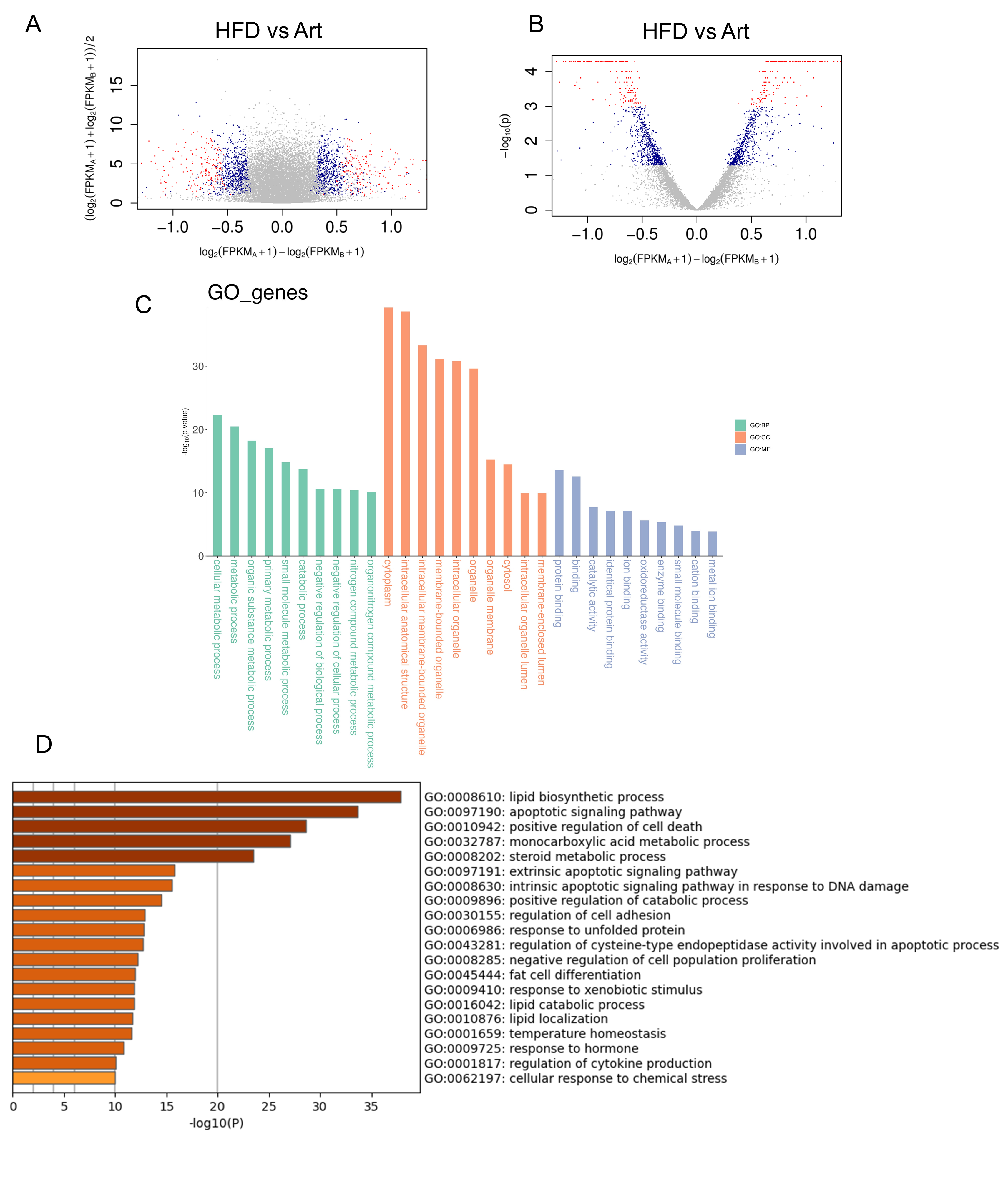

Supplement: Supplementary file 3 [file Image2.TIF]
