# Supplementary material for: Artemether Ameliorates Non-Alcoholic Steatohepatitis by Repressing Lipogenesis, Inflammation, and Fibrosis in Mice
Source: Front Pharmacol. 2022 May 2;13:851342. doi: 10.3389/fphar.2022.851342 (PMC9108288; doi:10.3389/fphar.2022.851342)
Supplement: Supplementary file 5 [file DataSheet1.docx]

Supplementary Material

# Supplementary Figures

## Supplementary Figures


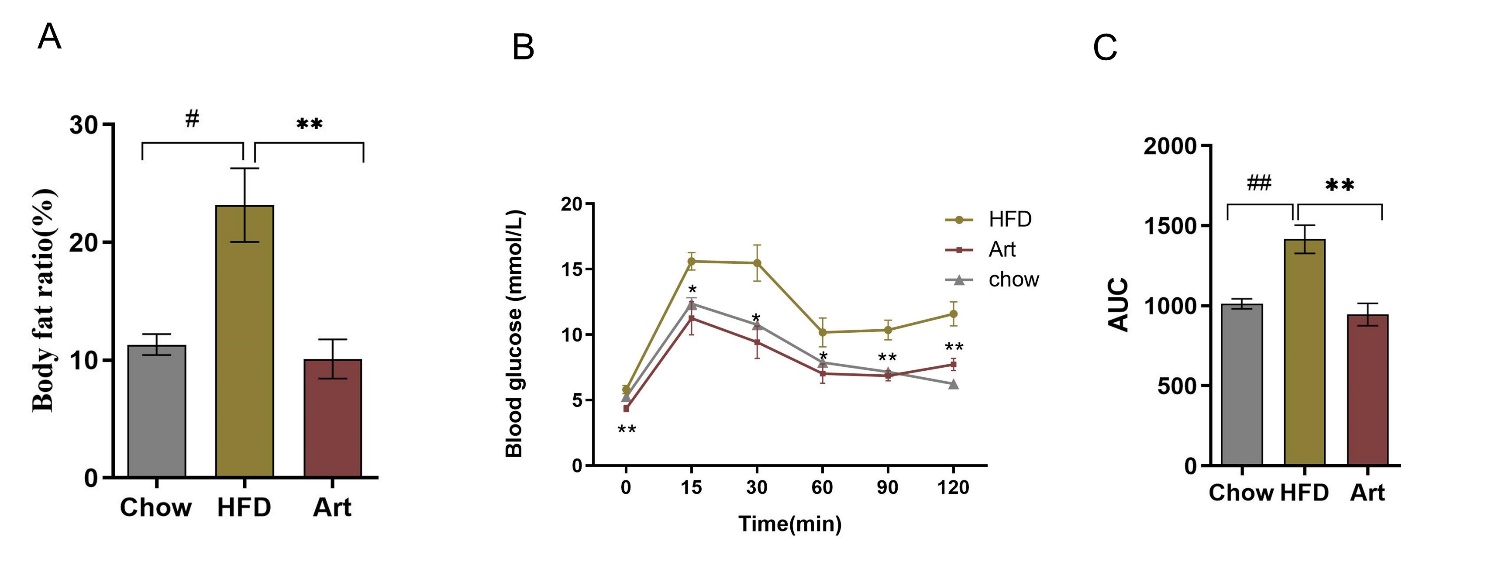


**Supplementary Figure 1.** Artemether improves NASH complications in a HFD diet induced mice. (A) Body fat rate. (B) GTT test, blood glucose level at 15, 30, 60, 90, 120 min after glucose injection. (C) GTT score result. Chow group vs HFD group: ^#^ *P* < 0.05, ^##^ *P* < 0.01 and ^###^*P* < 0.001. HFD group compared with Art group: * *P* < 0.05, ** *P* < 0.01 and *** *P* < 0.001.
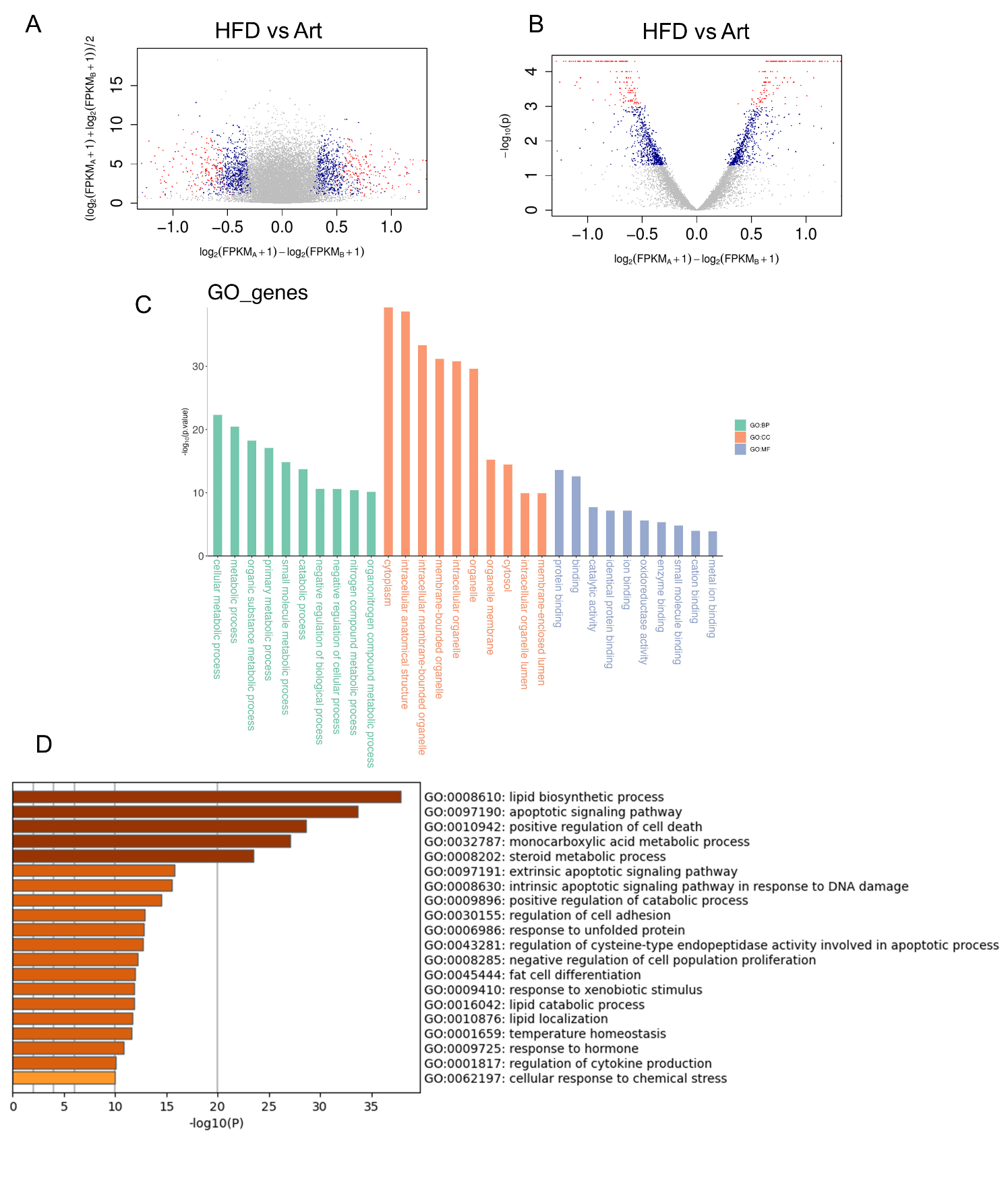


**Supplementary Figure 2.** The effect of Artemether treatment on liver lipid metabolic pathway in NASH Mice induced by an HFD Diet. (A) Volcano map. (B) MA-plot plots show the DEGs, blue indicates P < 0.05 and red indicates P < 0.01. (C) GO enrichment analysis of DEGs. (D) KEGG enrichment analysis of the DEGs significantly decreased in the Art group.

**
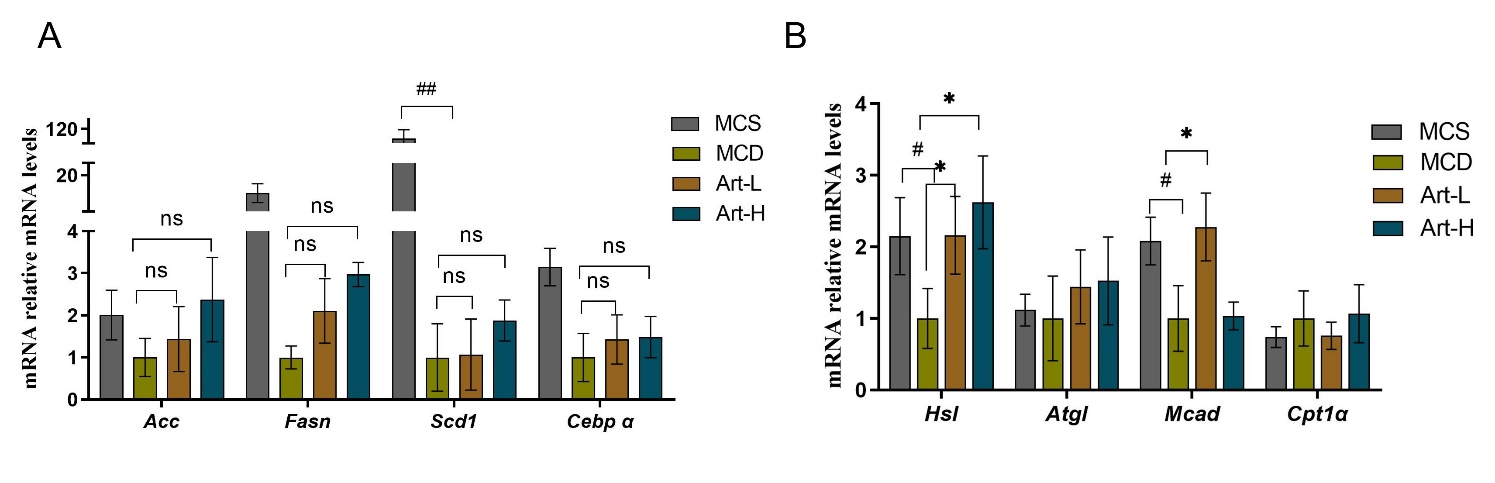
**

**Supplementary Figure 3.** Artemether improves lipid metabolism gene expression in MCD mice. (A-B) lipid synthesis-related genes (A) and lipolysis-related genes (B) expression in the liver was detected through Real-time PCR. * *P* < 0.05, ** *P* < 0.01and *** *P* < 0.001.

# Supplementary Table

Table S1: Primers used for qPCR

| Primer | Forward （ 5'to 3' ） | Reverse （ 5'to 3' ） |
| --- | --- | --- |
| *Mouse-Acc* | AGCTGATCCTGCGAACCT | GCCAAGCGGATGTAAACT |
| *Mouse-Acta 2* | GTTCAGTGGTGCCTCTGTCA | ACTGGGACGACATGGAAAAG |
| *Mouse-Timp1* | AGGTGGTCTCGTTGATTTCT | GTAAGGCCTGTAGCTGTGCC |
| *Mouse-Smad2* | GCCCCAACTGTAACCAGAGA | GAGCCAGAAGAGCAGCAAAT |
| *Mouse-Scd1* | GAGGGCTTCCACAACTACCA | AAACAGGAACTCAGAAGCCCAA |
| *Mouse -Cpt1α* | GACTCCGCTCGCTCATTCC | GACTGTGAACTGGAAGGCCA |
| *Mouse -Atgl* | CCAACACCAGCATCCAGT | CAGCGGCAGAGTATAGGG |
| *Mouse -Fasn* | TCCAAGACTGACTCGGCTACTGAC | GCAGCCAGGTTCGGAATGCTATC |
| *Mouse -Acsl1* | TGGGGTGGAAATCATCAGCC | CACAGCATTACACACTGTACAACGG |
| *Mouse -Srebp-1 c* | CCAGAGGGTGAGCCTGACAA | AGCCTCTGCAATTTCCAGATCT |
| *Mouse -Tgf-β* | CTTGTGACAGCAAAGATAATGTACT | TCCAACCCAGGTCCTTCCTAA |
| *Mouse -Il-1 β* | GAAATGCCACCTTTTGACAGTG | TGGATGCTCTCATCAGGACAG |
| *Mouse -Tnf-α* | AGACCCTCACACTCAGATCA | TCTTTGAGATCCATGCCGTTG |
| *Mouse -Mcp1* | AGATGCAGTTAACGCCCCAC | TGTCTGGACCCATTCCTTCTTG |
| *Mouse -Cxcl2* | CCAACCACCAGGCTACAGG | GCGTCACACTCAAGCTCTG |
| *Mouse -Cxcl10* | AAGTGCTGCCGTCATTTCT | GTGGCAATGATCTCAACACG |
| *Mouse -Il-10* | CTTACTGACTGGCATGAGGATCA | GCAGCTCTAGGAGCATGTGG |
| *Mouse -Cd11 c* | TGTTGGCGGAAGCAAATGG | GGGCAGGTTCAAAGAAGATGG |
| *Mouse -Cd197* | ACCTGGTTATCATCCGCACTC | CTGGAAGACGACGAACACTA |
| *Mouse -Cd206* | GAGGGAAGCGAGAGATTATGGA | GCCTGATGCCAGGTTAAAGCA |
| *Mouse -Cd163* | CTGGCGGGTGGTGAAAACA | CAGCCGTTACTGCACACTG |
| *Mouse -Arg-1* | CTCCAAGCCAAAGTCCTTAGAG | GGAGCTGTCATTAGGGACATCA |
| *Mouse -Ccl4* | TTCCTGCTGTTTCTCTTACACCT | CTGTCTGCCTCTTTTGGTCAG |
| *Mouse -Ccl2* | TTAAAAACCTGGATCGGAACCAA | GCATTAGCTTCAGATTTACGGGT |
| *Mouse -Ifnγ* | ATGAACGCTACACACTGCATC | CCATCCTTTTGCCAGTTCCTC |
| *Human-Acc* | GCATGGACGGGTACATCTTCAA | GTGTGACCATGACAACGAATCT |
| *Human-Fasn* | AAGGACCTGTCTAGGTTTGATGC | TGGCTTCATAGGTGACTTCCA |
| *Human-Scd1* | TCTAGCTCCTATACCACCACCA | TCGTCTCCAACTTATCTCCTCC |
| *Human-Srebp1c* | ACAGTGACTTCCCTGGCCTAT | GCATGGACGGGTACATCTTCAA |
| *Human-Cpt1* | TCCAGTTGGCTTATCGTGGTG | TCCAGAGTCCGATTGATTTTTGC |
| *Human-Atgl* | ACCTCAATGAACTTGGCACC | CAACGCCACGCACATCTA |
